# Trusted Autonomous Operations of Distributed Satellite Systems Using Optical Sensors †

**DOI:** 10.3390/s23063344

**Published:** 2023-03-22

**Authors:** Kathiravan Thangavel, Dario Spiller, Roberto Sabatini, Stefania Amici, Nicolas Longepe, Pablo Servidia, Pier Marzocca, Haytham Fayek, Luigi Ansalone

**Affiliations:** 1 Sir Lawrence Wackett Defence & Aerospace Centre, RMIT University, Melbourne, VIC 3000, Australia; 2 School of Aerospace Engineering, Sapienza University of Rome, 00138 Rome, Italy; 3 Department of Aerospace Engineering, Khalifa University of Science and Technology, Abu Dhabi P.O. Box 127788, United Arab Emirates; 4 National Institute of Geophysics and Volcanology (INGV), 00143 Rome, Italy; 5 Φ-Lab Explore Office, ESRIN, European Space Agency, 00044 Frascati, Italy; 6 Comisión Nacional de Actividades Espaciales (CONAE), Av. Paseo Colón 751, Buenos Aires C1063ACH, Argentina; 7 School of Engineering, RMIT University, Melbourne, VIC 3000, Australia; 8 School of Computing Technologies, RMIT University, Melbourne, VIC 3083, Australia; 9 Engineering and Future Space Systems Unit, Italian Space Agency, 00198 Rome, Italy; 10 SmartSat Cooperative Research Centre, North Terrace, Adelaide, SA 5000, Australia

**Keywords:** astrionics, bushfire, disaster management, distributed satellite systems (DSSs), edge computing, hyperspectral imagery, intelligent DSS (iDSS), mission management, optical sensors, PRISMA, trusted autonomous satellite operations (TASO), wildfire

## Abstract

Recent developments in Distributed Satellite Systems (DSS) have undoubtedly increased mission value due to the ability to reconfigure the spacecraft cluster/formation and incrementally add new or update older satellites in the formation. These features provide inherent benefits, such as increased mission effectiveness, multi-mission capabilities, design flexibility, and so on. Trusted Autonomous Satellite Operation (TASO) are possible owing to the predictive and reactive integrity features offered by Artificial Intelligence (AI), including both on-board satellites and in the ground control segments. To effectively monitor and manage time-critical events such as disaster relief missions, the DSS must be able to reconfigure autonomously. To achieve TASO, the DSS should have reconfiguration capability within the architecture and spacecraft should communicate with each other through an Inter-Satellite Link (ISL). Recent advances in AI, sensing, and computing technologies have resulted in the development of new promising concepts for the safe and efficient operation of the DSS. The combination of these technologies enables trusted autonomy in intelligent DSS (iDSS) operations, allowing for a more responsive and resilient approach to Space Mission Management (SMM) in terms of data collection and processing, especially when using state-of-the-art optical sensors. This research looks into the potential applications of iDSS by proposing a constellation of satellites in Low Earth Orbit (LEO) for near-real-time wildfire management. For spacecraft to continuously monitor Areas of Interest (AOI) in a dynamically changing environment, satellite missions must have extensive coverage, revisit intervals, and reconfiguration capability that iDSS can offer. Our recent work demonstrated the feasibility of AI-based data processing using state-of-the-art on-board astrionics hardware accelerators. Based on these initial results, AI-based software has been successively developed for wildfire detection on-board iDSS satellites. To demonstrate the applicability of the proposed iDSS architecture, simulation case studies are performed considering different geographic locations.

## 1. Introduction

Low-cost Distributed Satellite Systems (DSS) will potentially play a significant role in the design of future space missions. These systems will collaborate to accomplish difficult mission goals. Real-time multi-spacecraft coordination, data processing, and prioritisation will not only optimise mission science return by establishing observational parameters of interest or success, but it will also facilitate outer solar system missions and missions in extreme environments (e.g., Io, Venus, subsurface Europa) where communication with ground operations and ground-based analysis times are limited. This will allow for a greater return on investment in mission science. This capability will enable previously unthinkable classes of missions by providing levels of autonomy that are unparalleled in the sector [1,2,3]. It will be necessary to make significant advances in the capabilities of the architectures that are used to implement these envisioned space missions in order to be able to carry them out. DSSs are comprised of a large number of spacecraft that cooperate with one another to accomplish a specific mission objective [4]. In some circumstances, DSSs combine to generate a sensory system that would be impossible to create on a monolithic platform [5,6]. In other configurations, they use distributed measurements to extract data on the spatial and temporal consequences of phenomena that are far larger than what a single spacecraft can observe due to limited swath width and duty cycle [7,8].

There has been a great deal of interest in addressing the technology required to enable new applications, particularly in cases where DSS-dependent missions are becoming increasingly important. On-board processing, inter-satellite networks, and autonomous decision making are the primary focuses of this article. These three technologies are interdependent and cannot exist without one another. For example, the processors that are implemented on board must have sufficient processing capability in order to process the data that are necessary for drawing conclusions as well as any computation that may be involved in the process of making decisions that are time-sensitive or computationally intensive. In addition, owing to the inter-satellite networks, the DSS spacecraft are able to communicate with one another and coordinate their activities without the need for immediate ground control. Because the ground link has a restricted bandwidth and latency, this feature is critical, especially for activities that take place in outer space [9,10]. A mission’s risk is reduced when it is spread among several launches, ensuring that the entire system is not destroyed in the event of a launch failure. Additionally, it provides the option to gradually build the system in orbit, allowing for the construction of various modules at various stages. The modular architecture theory serves as the foundation for DSS architecture. A study by the Research and Development (RAND) Project air force shows that [11]:

(a) Distributed constellations may weigh less and cost less to launch.
(b) Distributed satellites may perform better during deployment.
(c) Distributed satellite constellations may be able to fail more gracefully.
(d) Distributed satellite constellations may be more survivable in a cyber attack.

**Figure 1:**
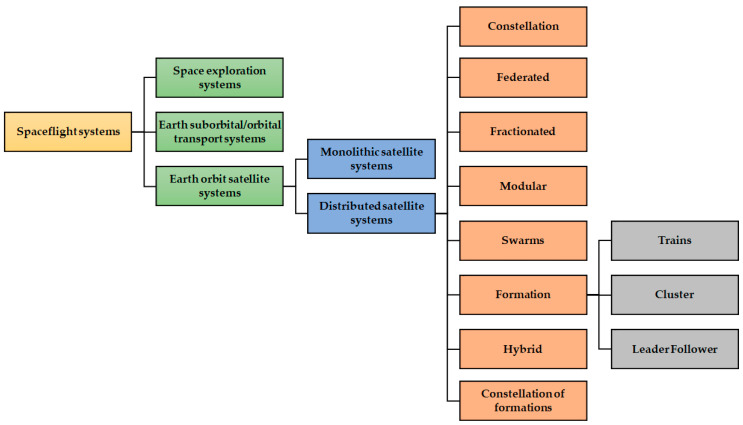
Classification of satellite systems.

The primary goal of a DSS is to deliver a more responsive and resilient solution to meet the expanding demands of the scientific community and also the defence sector by aiding in the measurement and prediction of Earth Observation (EO) missions [12] and Space Based Space Surveillance (SBSS) missions [13,14,15,16,17,18] in the hope of enhancing space sustainability. Distributed satellite architectures can also be classified as formation-flying missions. Examples include PRISMA [19,20], GRACE [21], and TerraSAR-X–TanDEM-X [22]. DSS are categorised based on the type of mission and function they perform. Activities required to meet local objectives (i.e., those specific to each module) or small bits of a global objective’s functioning (i.e., particular to the infrastructure) may be included in modules performing activities in a distributed infrastructure, whether it be in independent satellite systems or distributed spacecraft. The main classification of the satellite system is shown in Figure 1. Table 1 provides a detailed description of different types of DSS architecture. The level of the operational independence of a satellite or a fraction of distributed spacecraft is characterised as *operational/functional independence*. Individual spacecraft or fractions of a distributed spacecraft’s *homogeneity* is defined as the degree of similarity between them [9,23,24,25].

A DSS has several advantages: (i) simultaneous multipoint data collection, (ii) increased availability, (iii) the ability to look at different things at once, and (iv) reduced downtime and graceful degradation. A DSS that includes these enabling technologies can provide four major benefits [3]:

- *Distributed Coordination:* can share data and change what they prioritize.
- *Autonomous Re-tasking:* can respond to environmental stimuli autonomously, without requiring intervention from a ground operator.
- *Increased Availability:* when only a single spacecraft can be reached, it can relay commands to the others.
- *Workload Balancing:* can re-task satellites based on available computation, power, and communications resources.

The purpose of this research is to demonstrate the Trusted Autonomous Satellite Operations (TASO) of a DSS for mission management, such as wildfire detections. The findings of such analyses could be useful for future time-critical missions, i.e., disasters and rare events, and the following contributions were made from an intelligent DSS (iDSS) perspective:

- ***Mission Astrionics:*** A reactive element, such as Artificial Intelligence (AI), is integrated with the DSS to achieve TASO for on-board data processing to provide real-time/near-real-time alerts. To accomplish the same, deep learning is developed and demonstrated for detecting wildfires on-board the satellite using optical payload, i.e., hyperspectral imagery.
- ***Service Astrionics:*** For the TASO, the iDSS will reconfigure either based on the detection of a disaster event (wildfire) or based on the requirements of the owner/operator for the requested duration.

Due to the Inter-Satellite Link (ISL) between the satellites in the architectures, iDSS can execute TASO. It is essential to mention that the detection of wildfires should be treated as an example test case and that the suggested methodology (or ones similar to it) can be successfully applied to other scenarios or activities, as has already been explored and shown in other publications [26].

The article is organised as follows: following the introduction, Section 2 discusses a DSS and its operation. Section 3 discusses the reconfiguration and reactive element in the DSS, followed by more in-depth information about the study area’s description, and finally, information about the PRISMA data and the dataset’s definition as well as the on-boardimplementation are covered. Section 4 delves deeply into the results and findings, as well as their applicability, and is followed by a conclusion in Section 5. This is an extended version of an article [27] that was presented at the 2022 IEEE MetroXRAINE Conference.

## 2. Distributed Satellite Systems

**Figure 2:**
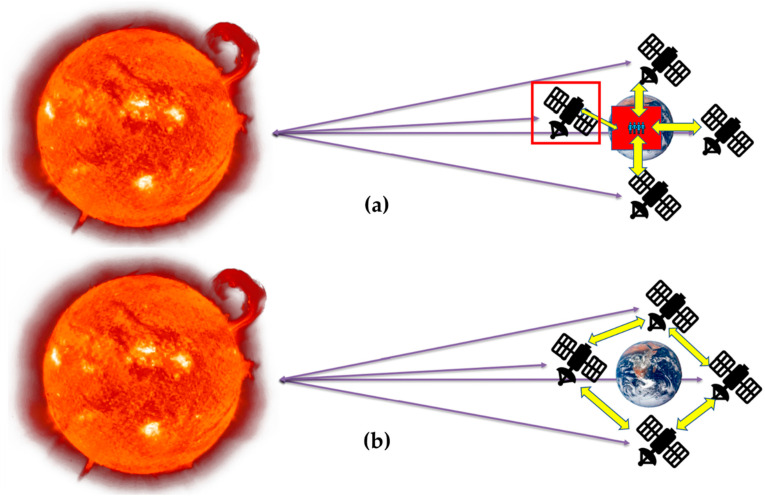
(**a**) Current state-of-the-art DSS operations; (**b**) DSS operation with ISL, i.e., iDSS. Adapted from [3].

The current state-of-the-art DSS operation is completed when one of the satellites picks/detects the event, then it is sent to ground control, and the ground control operators conduct the reconfiguration, which is inefficient in time-critical applications. Figure 2a depicts the current state of the DSS. As shown, the highlighted satellite detects the event and transmits it to the ground station, where it is relayed to the remaining DSS satellites. With the ISL, the DSS operation can be improved, and real-time/near-real-time operations such as data sharing/processing can be performed without the ground segment as shown in Figure 2b. This provides enhanced performance for time-critical applications such as a rare event, disaster events, etc. With the ISL and the DSS, real-time operations are achieved by adding predictive and reactive elements [28,29,30] within the architecture that endows the iDSS.

**Figure 3:**
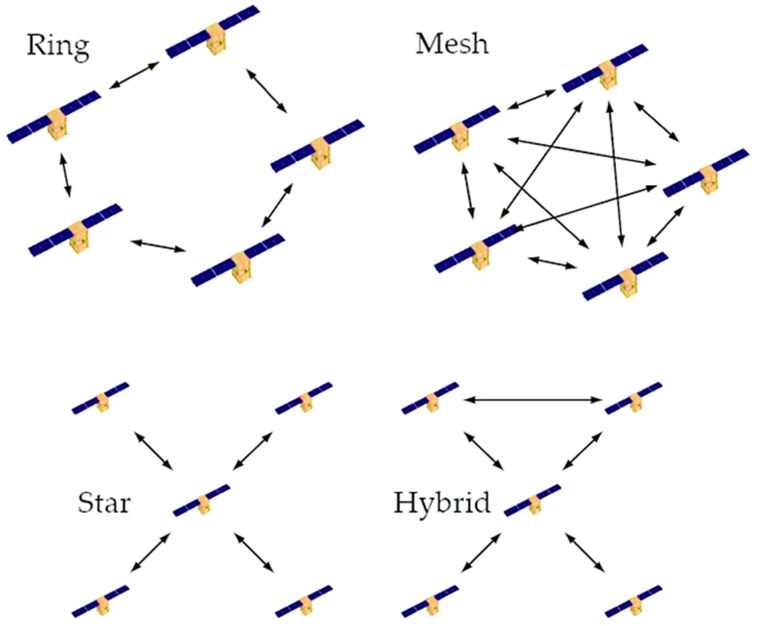
ISL classification [31].

**Figure 4:**
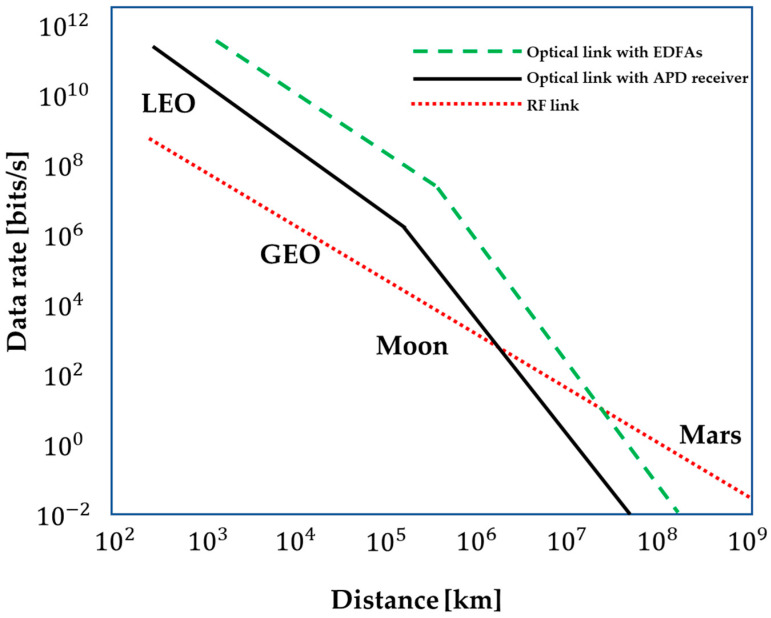
Link distance against data rates for optical and RF ISL systems. Adapted from [36].

The DSS can communicate, interact, and cooperate with the ISL. ISL makes up for the lack of robustness in the DSS, which results in an increase in the amount of data exchanged and communication that takes place on-board the satellite in the DSS. Liz Martinez et al. [31] provide the various strategies that are suited for DSSs. ISL can be classified as a (1) ring, (2) star, (3) mesh, and (4) hybrid configuration depending on the communication linkages that are established between the DSS. These topologies are depicted in Figure 3, with the ISL represented by the arrows. Liz Martinez et al. provide a wide range of solutions in their article [31] that are ideal for DSSs. Communication by radio frequency, also known as RF, is a form of transmission that is used in wireless networks more commonly than any other method. However, current space optical communication systems promise a bigger number of benefits, such as an improved data rate, protection, lower power consumption, and a decrease in the weight of satellites. In the past, RF technology was used for inter-satellite communication; however, these days, modern satellites are increasingly resorting to technology that is based on laser and optics in order to connect with one another. Utilizing technologies that are based on lasers comes with a multitude of advantages. To begin, infrared laser rays have a greater frequency when compared with RF, which results in a shorter wavelength. As a direct consequence of this, they are able to send a greater quantity of data in a single transmission. Second, in contrast with radio waves, lasers experience significantly less difficulty with dispersion when they are transmitted over extensive distances. Because of this, it is far more difficult to intercept them, which results in a large increase in the level of security guaranteed to the data transfer [32,33,34]. Figure 4 illustrates the applicability of RF and optical communication (using two versions, i.e., Avalanche Photodiode (APD) and Erbium-Doped Fibre Amplifiers (EDFAs)) by plotting data rate against distance. Laser-based mesh topologies are excellent for the iDSS operations due to the fact that they are more dependable and are well-suited for use in applications that require real-time processing [35].

**Figure 5:**
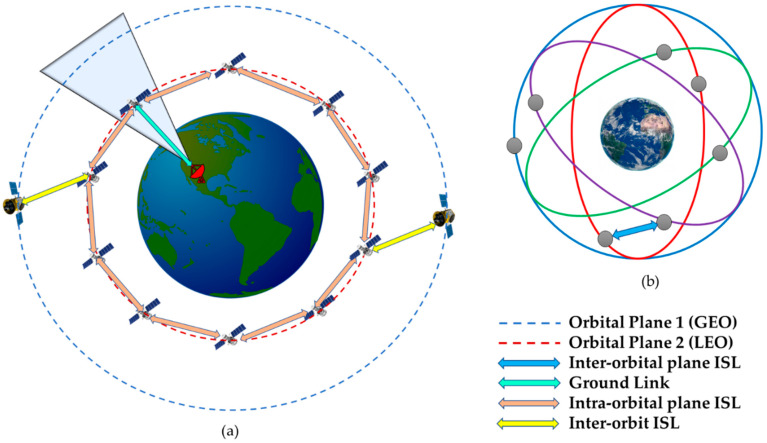
(**a**) ISL relationship between the orbits and the ground station; (**b**) proposed iDSS constellation with ISL.

The DSS collaborates actively through the ISL where the information is shared to achieve a common mission objective. The ISL relationship between the ground station network, with the iDSS orbital plane and other orbits, is shown in Figure 5. Figure 5a shows the ISL relationship between the orbits and the ground segment, whereas Figure 5b shows the proposed iDSS constellation with ISL.

**Figure 6:**
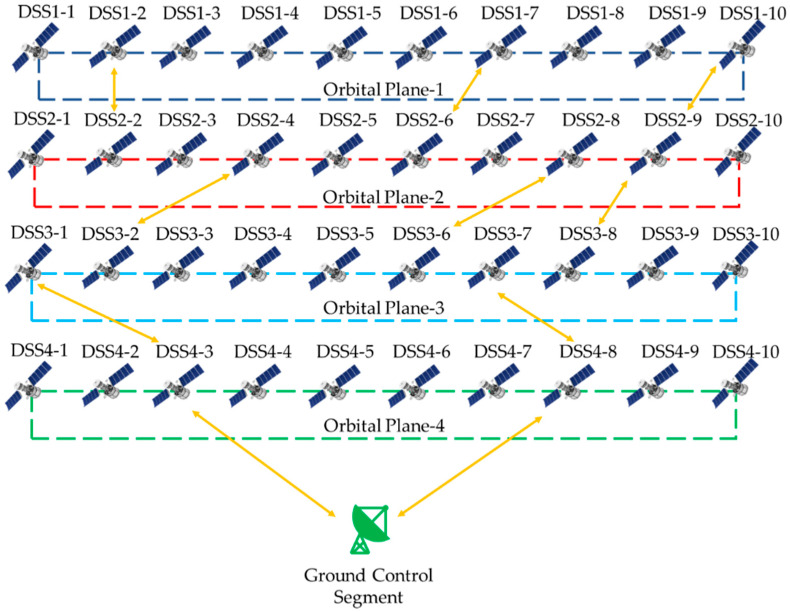
An instance of the proposed EO constellation illustration with inter-orbital plane ISL and ground link.

This research work establishes a constellation of Low Earth orbit (LEO) satellites [37,38,39,40,41,42] for EO [43] disaster event management such as droughts [44], sandstorms [45], rising sea levels [46], tornados [47], volcanic eruption [48], wildfires [49], etc. In particular, wildfire is chosen because one of the Sustainable Development Goals (SDG:13) is climate action, and among the primary catastrophe events that have an impact on climate is wildfire. In Australia and other countries, large-scale forest fires have dramatically grown in rate of recurrence and size in recent years. In the past 15 years, there have been 18 wildfire events in Australia [50]. For the same, an iDSS, i.e., a constellation of satellites, is proposed as shown in Figure 6 with ISL to provide near-real-time disaster management.

**Coverage Analysis:** An imprint in the shape of a circle or rectangle is left on the surface of the Earth whenever a satellite is used to observe a particular area. The separation between the spacecraft and a targeted point within the satellite’s field of view region (also known as the imprint region) at a specific point in time is referred to as the instantaneous coverage of the satellite. The value of the Earth central angle λ is determined by the following equation where $\left(\Theta_{s},{\;\Lambda }_{s}\right),$ denotes the latitudes and longitudes of the sub-satellite point and $\left(\Theta_{t},{\;\Lambda }_{t}\right)$ denotes the latitudes and longitudes of the target [51].

(1) $$\cos\lambda \;={\;\sin\Theta }_{s}{\sin\Theta }_{t}+{\cos\Theta }_{s}{\cos\Theta }_{t}\cos\left|\Lambda_{s}-\Lambda_{t}\right|\;$$

Then, the nadir η is computed, which is then utilised to obtain the maximum Earth central angle $\lambda_{\max}$.

(2) $${\mathrm{Sin}\;\eta }_{\max}=\cos\;\epsilon_{\min}\left[\frac{R_{E}}{R_{E}+h}\right]\;$$

(3) $$\lambda_{\max}=90{}^{\circ}-\epsilon_{\min}-{\;\eta }_{\max}$$

**System-Wide Access:** It is essential to determine the period that at least one spacecraft’s camera can perceive the Area of Interest (AOI) during this timeframe in order to compute the coverage and system-wide access. The corresponding percentage quantity is referred to as the system-wide access percentage, and it is calculated by using the following equations:

(4) $$\mathrm{SWAD}=n\cdot S_{c}\;$$

(5) $$\mathrm{SD}={\;\mathrm{ST}}_{\mathrm{Start}}-{\mathrm{ST}}_{\mathrm{Stop}}$$

(6) $$\mathrm{SWAP}=\frac{\mathrm{SWAD}}{\mathrm{SD}}\cdot 100\;$$

where SWAD stands for “system wide access duration”, n represents the number of elements in the system-wide access status whose value is “true”, i.e., 1, which means that the satellite has a full view of the AOI; $S_{c}$ denotes the sampling time of the spacecraft; and SWAP stands for “system wide access percentage”. The above-mentioned equations, which relate to the nadir pointing, can also be applied to systems that have reconfiguration capabilities:

(7) $${\mathrm{SWAD}}_{T}=N\cdot S_{c}\;$$

(8) $$\mathrm{SD}={\;\mathrm{ST}}_{\mathrm{Start}}-{\mathrm{ST}}_{\mathrm{Stop}}$$

(9) $${\mathrm{SWAP}}_{T}=\frac{{\mathrm{SWAD}}_{T}}{\mathrm{SD}}\cdot 100\;$$

where ${\mathrm{SWAD}}_{T}$ is the system-wide access duration with reconfiguration; N is the number of elements in the system-wide access status with reconfiguration whose value is true, which means the satellite has full view of the AOI; $S_{c}$ is the spacecraft sample time, which is considered to be 30 s for both of these scenarios; and ${\mathrm{SWAP}}_{T}$ is the system-wide access percentage with reconfiguration [35].

## 3. Reactive Features and Reconfiguration in iDSS

In order to process the data on-board the satellite, a reactive element, such as on-board processing with AI, is required [52,53,54,55]. In our earlier works [55,56,57,58,59,60], we demonstrated the viability of AI-on-the-edge paradigms. A one-dimensional (1D) convolutional neural network (CNN) was explored for observing bushfires on-board the satellite employing optical sensors, and encouraging results for the edge implementation on three different hardware accelerators were demonstrated. There are various optical sensors that have been deployed and used for a variety of purposes in Earth’s orbit, and the main important optical characteristics are detailed in Table 2. PRISMA hyperspectral imagery data from the listed optical sensor was used to demonstrate the on-board processing capabilities. The VNIR channel, in particular, is used for wildfire management.

**Figure 7:**
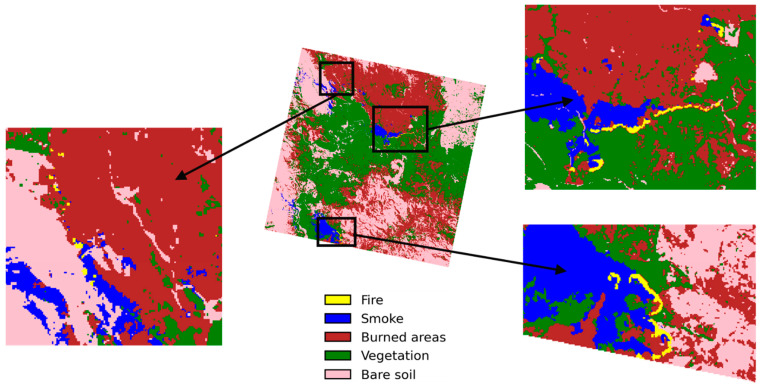
Wildfire segmentation map of the hyperspectral imagery over Australia [56].

The simulation made use of the PRISMA optical sensor, i.e., hyperspectral imagery, and it was shown that AI-on-the-edge paradigms for futuristic mission proposals are feasible by leveraging proper CNN architectures and established technology to execute time- and power-efficient inferences. The analysis was carried out using Level 2D data. It is important to point out that we directly employed high-level products in this instance, specifically L2D, and assumed that the pre-processing is carried out on board prior to the analysis due to the fact that the pre-processing demands a significant amount of time for the fine band-to-band alignment, the fine georeferencing, the radiometric calibration, and the radiance to reflectance conversion. We were able to gather optical imagery of wildfires over Australia and used the same for analysis. Figure 7 presents the segmentation image of the study region with three different fires over Australia. Nevertheless, by focusing on individual bands, one can extract specific information. The strategy of using AI is utilised in order to put into action automatic segmentation from the image that was collected using the optical payload.

**Figure 8:**
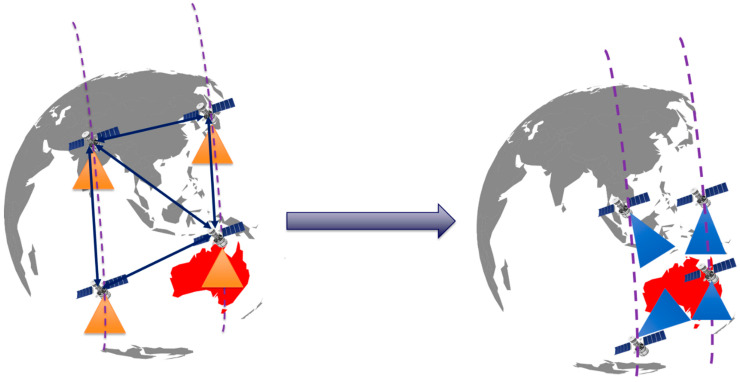
An illustrative view of iDSS reconfiguration.

The requirements of the owner or operator can be used to customise the iDSS operations. In the event that real-time mission management is required, the iDSS will reconfigure autonomously. The proposed iDSS makes use of a constellation of forty satellites, all of which are continuously connected to one another through ISL. The proposed iDSS is assumed to be in near-circular orbit (i.e., eccentricity is 0.001), 500 km altitude, and inclination 55, with 40 satellites evenly spaced (plane spacing 36) in 4 orbital planes. We can disregard the values of the Right Ascension of the Ascending Node (RAAN) and mean anomaly because we are considering a continuous coverage problem. The proposed constellation’s participants are assumed to be similar and to carry the same optical payload [35]. In general, every satellite will be in periodic planning, and the nadir orientation of the camera will be maintained at all times. If one of the satellites in the constellation detects the event, such as a wildfire, then that satellite will communicate with the other satellites in the constellation. The primary objective at that point will be to acquire as much imagery as possible and to process the data on board using astrionics, i.e., hardware accelerators. After that, the data that can be acted upon is transmitted to the owners and operators. Once the wildfire is identified, the spacecraft would use the active Attitude and Orbit Control System (AOCS) for real-time/near-real-time disaster event management, as illustrated in Figure 8, with a limit concerning the off-nadir pointing ensuring sufficient image quality. It should be noted that in this case, attitude change is only considered for payload reconfiguration, which is accomplished using a gimbal. Because LEO is densely populated, an active orbital control is used by means of an electric thruster in the event of a collision between satellites [10,29,63].

## 4. Results and Discussion

Since the end goal is to create a model that can be uploaded to an on-board astrionics system in the iDSS, it is necessary to optimise the network complexity, parameter count, and inference processing time. The use of a small chip reduced the capability to execute the wildfire detection task, which necessitated the development of an accurate model as a result of the hardware’s constraint. In order to assess the viability of the suggested methodology, a prototype for carrying out the study was developed. To get started, the model was altered so that it is compatible with the hardware that was selected and so that it can detect wildfires on-board the iDSS. The model is trained with the assistance of the sophisticated computer on the ground, and the trained model is evaluated in astrionics, i.e., the hardware accelerators for the on-board data processing that are detailed in Table 3.

From the reported results for the CubeSats or other small satellites, the Movidius and the Jetson Nano appear to be the most promising possibilities since they are in line with the entire power budget of the spacecraft platform. All of the findings that have been reported in Table 3 are in line with this budget. In this particular instance, we are taking into consideration the presence of Jetson Nano and Intel Movidius on-board the proposed constellation for the detection of wildfires. When it comes to CubeSats or small satellites, the Movidius, which has an inference time (inference time is the amount of time it takes for a model to process data and make a prediction) of 5.8 ms and a power consumption of 1.4 W, and the Jetson Nano, which has an inference time of 3.4 ms and a power consumption of 2.6 W (2.0 GPU only), appear to be the most promising options. These findings come from our previous work [56,57,58]. All of the results obtained are in line with the spacecraft platform’s total power budget. The introduction of reactive features into the DSS architecture enables the TASO, as demonstrated by the results presented above.

**Figure 9:**
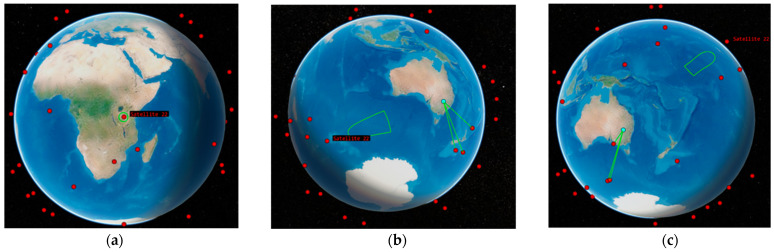
Satellite Field of View: (**a**) nadir pointing; (**b**) reconfiguration at the entry; (**c**) reconfiguration at the exit [35].

An analysis of the AOI on the Earth and conical sensors on-board a heterogeneous constellation of satellites is presented as part of the simulation. If a ground station is located in the Field of View (FOV) of a satellite’s conical sensor and in the Elevation Angle (EA) of the conical sensor with regard to the AOI, then it is said that the AOI and the conical sensor have access to the ground station. For the purpose of simulation, the altitude of the KANYINI mission in considered, as presented before the simulation makes use of a constellation of 40 low-Earth orbiting satellites located at an altitude of 500 km. To generalise the results of the analysis, the likelihood of wildfires occurring on the four continents, i.e., four different AOI, was carefully selected. Every satellite has a camera with a FOV of thirty degrees, and the mission of the entire satellite network is to acquire images of the AOI whenever there is adequate illumination from the sun. In order to gather high-quality pictures with minimal effects of atmospheric distortion, the Earth-orbiting satellite’s EA should be at least 30 degrees with respect to the AOI. It is important to compute the windows of time during a predetermined period of six hours during which each satellite can obtain an image of the location in consideration. It is also important to compute the percentage of time that a camera on at least one satellite could see the location throughout this duration. This percentage is given by SWAP and be calculated based on Equation (6). The fact that the AOI may be found inside the contour provides evidence that it is visible in the FOV of the payload camera. The FOV of the satellite’s visualisation is depicted in Figure 9a for the nadir-pointing configuration. In addition to calculating the times when each camera can capture the AOI, it is necessary to determine the system-wide access percentage, which corresponds to the percentage of time from the simulation start time to the stop time during which at least one satellite can observe the AOI. This must be done before the system-wide access percentage can be calculated [35].

The nadir-pointing orientation is the default attitude arrangement for the satellite. The cameras always aim straight down because they are aligned by default with the yaw axis; hence, the AOI is therefore no longer accessible to the cameras until their EA dips below 30 degrees. As a direct consequence of this, the FOV of the cameras limits the cumulative access percentage. On the other hand, if the DSS is able to connect with the ISL and have reactive components in the architecture, then the satellites will be able to communicate with the satellites that are positioned close to them. The cameras on the satellites will then be adjusted such that they are constantly directed in the direction of the AOI using active attitude control; the AOI will be observed as long as the Earth does not get in the way, as shown in Figure 9b,c, respectively. As a direct consequence of this, the percentage of system-wide access will now be constrained, not by the camera’s field of view but rather by the AOI’s minimum effective area. This is carried out according to the requirements that the owner/operator has for the time period that was requested. When the AOI moved into or out of the field of view of the camera, the access periods for that scenario began and ended at the appropriate times. To be more specific, it enters the FOV after the camera’s EA has been greater than 30 degrees and leaves the FOV before the EA has been reduced to less than 30 degrees. The remaining time is spent with the camera oriented in the direction of the nadir.

- Australia

**Figure 10:**
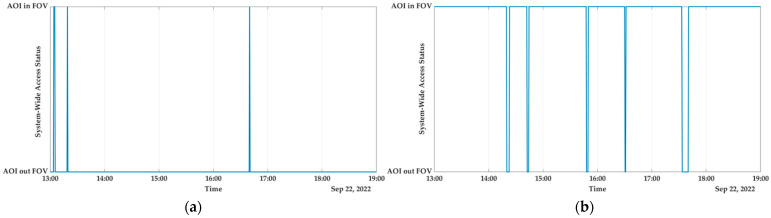
Australia: (**a**) system-wide access status; (**b**) system-wide access status with reconfiguration.

Due to its climate, topography, and vegetation, Australia is susceptible to large-scale wildfires due to the interaction of all three factors. In recent years, there have been multiple instances of wildfires breaking out. A region in New South Wales that has a high risk of being affected by wildfires has been taken into consideration, and the analysis is currently taking place [50]. According to the initial findings of the investigation, the computed reconfiguration coverage for the Australian AOI is 95.9722%. This represents a coverage level that is nearly equivalent to real time for the monitoring of catastrophic events. Examining Figure 10, which depicts the system with access status being given for both the (a) nadir configuration and (b) the reconfiguration, this result can be comprehended in a more clear and concise manner. In the second scenario, the AOI is hidden from view for admittedly very short periods of time which endows the real-time/near-real-time monitoring.

b. Africa

**Figure 11:**
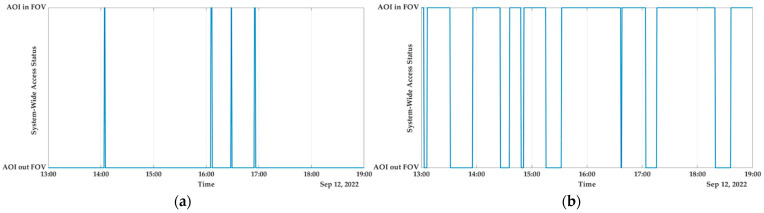
Africa: (**a**) system-wide access status; (**b**) system-wide access status with reconfiguration.

In southern Africa, drier conditions have become more pronounced over the years, which has led to an increase in wildfire occurrence and extreme drought conditions. These conditions, either on their own or in combination, have led to a loss of crop productivity, the deaths of livestock and other wildlife, famine, and the degradation of ecosystems, as well as a reduction in water quality and quantity. It is anticipated that there will be a 5.4% rise in the annual burned area throughout southern Africa in particular. These conditions, which are typical of southern Africa with their large variations in rainfall and regular droughts, make the arid and semi-arid regions more prone to the outbreak of wildfires [64]. Taking all of these factors into account, Angola is factored into our analysis and the results are shown in Figure 11. In conclusion, there is a decrease in performance in the African site, which reflects a value for ${\mathrm{SWAP}}_{T}$ that is 75.6944%. This is due to the geographical position of Angola on the globe (the distance between spacecraft is maximum when close to the equator).

c. Europe

**Figure 12:**
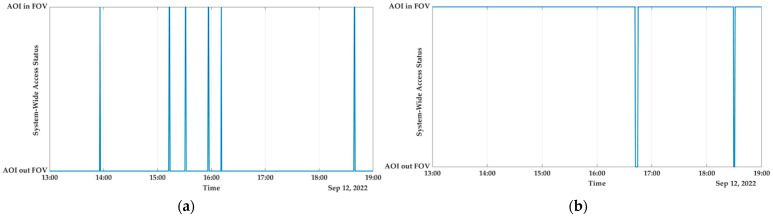
Italy: (**a**) system-wide access status; (**b**) system-wide access status with reconfiguration.

In Europe, the island of Sardinia, which is located in Italy, is home to a significant number of urban interfaces, recreational values, and highly valued agricultural areas, all of which are in danger of being destroyed by severe wildfires due to the island’s large population density. The majority of the fires that occur on these islands are started by individuals and can be traced back to human negligence, agricultural and pastoral land use, as well as intentional arson. Based on the collected data from 1995 to 2009, the island of Sardinia has an annual average of 2219 fires, and the size of each fire is on average 7 ha. Each year, wildfires consume an area that is equivalent to 16,601 hectares on average, with the largest fire ever recorded consuming 9029 hectares. While fires larger than 50 ha make up only 1.8% of all fires (or about 40 per year), they are responsible for 68.7% of the total annual area that is burned [65,66]. In accordance with the results of the simulation as shown in Figure 12, with the capability of reconfiguration, the ${\mathrm{SWAP}}_{T}$ percentage is 98.889%, which provides real-time/near-real-time monitoring over the region in the event that a wildfire is detected.

d. North America

**Figure 13:**
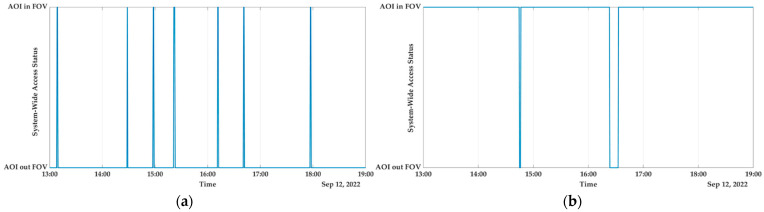
USA: (**a**) system-wide access status; (**b**) system-wide access status with reconfiguration.

The western United States is facing a growing threat from wildfires fuelled by synergies between historical fire suppression efforts, shifting land use, insects and disease, and climate shifts that are becoming drier and warmer. In the United States, wildfires, which are the most significant form of natural disturbance in temperate forest ecosystems, affect an average of 4500 km^2^ each year. Communities and land managers in areas at risk of wildfire have an immediate need for mitigation strategies to lower the likelihood of wildfires and adaptation strategies to improve the resilience of ecosystems in the face of changing weather patterns and fire patterns. One of the regions that has been impacted is the rugged terrain of north-east Oregon, whose economies have traditionally been dependent on the region’s forests and other natural resources. For the purpose of this research work, the Oregon region has been chosen, and after conducting the analysis, we determined that the ${\mathrm{SWAP}}_{T}$ is 97.0833% as shown in Figure 13, which guarantees continuous coverage over that AOI.

Table 4 displays, for the purposes of nadir pointing and reconfiguration, the system-wide access that is available for four distinct AOI. From Figure 10, Figure 11, Figure 12 and Figure 13, one can see how drastically different the two scenarios are from one another. The latitude and longitude coordinates of the four AOI that were chosen are presented in World Geodetic System (WGS84) format. The full simulation was run for a duration of 21,600 s, which is equivalent to 6 hours, and the results of the respective system-wide access with nadir pointing and with reconfiguration are provided. Because the cameras are firmly attached to the satellites, each satellite needs to be continually reoriented (i.e., manipulated using the on-board actuators) along its orbit such that its yaw axis tracks the location of the area of interest (AOI).

When the active AOI pointing is carried out using the AOCS on-board the satellite, the SWAP is significantly increased and provides almost real-time or near-real-time coverage, i.e., 75% to 98%, which will facilitate the near-real-time disaster response. This is evident from the results that were reported, and it is clear that when the nadir pointing is used, the FOV is relatively low for most AOI.

**Figure 14:**
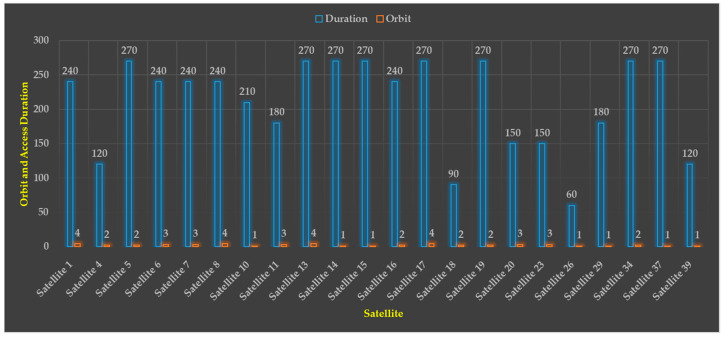
Australia satellite access duration with reconfiguration and its orbit.

Figure 14 shows the list of spacecrafts that will access the AOI as part of the planned constellation. It also displays the amount of time that these satellites will have access to Australia, and it presents the orbits that correspond to these satellites throughout the duration of the simulation time. These findings suggest that astrionics, i.e., hardware accelerators for on-board edge computing, could be considered for future space missions. This would allow for the improvement of the framework, the efficient organisation of space-to-ground dataflow, and the provision of real-time or near-real-time information, both of which could be very helpful in the management of extreme events and humanitarian emergencies. It was discovered that the outcomes were influenced by the direction in which the sensors were pointed when the measurements were taken. These results can also be affected by the orbits of the satellite, the minimum EA of the AOI, the camera mounting position, and location in respect to the FOV of the satellite if the satellite is not continually pointed at the AOI.

The orbits of the satellites can be altered by employing Keplerian parameters and by modifying to the appropriate AOI in accordance with the needs of the owner or operator. In the future, cameras will be able to be mounted on gimbals that can rotate freely on the satellite, and the many sensors that are distributed across the constellation will be able to be used to improve the results. This not only makes it possible for the satellites to point directly downward, also known as nadir pointing, but it also makes it possible for the gimbals to be adjusted so that they can track the AOI independently, and it makes it possible for heterogeneous sensors to provide useful data at a diverse array of wavelengths.

## 5. Conclusions and Future Work

The application of Trusted Autonomous Satellite Operations (TASO) is feasible in Distributed Satellite Systems (DSS) owing to the incorporation of reactive elements in the management of various types of Earth Observation (EO) missions. The applicability to a wildfire sensing and management mission is demonstrated in this research work, focusing on real-time/near-real-time operations. Since the results are promising, the same approach can be used for other EO/disaster management events such as flood detection, hazardous zone monitoring, and volcanic eruption. This is made possible by the DSS’s autonomous reconfiguration and predictive/reactive integrity features, which can be provided by AI-based software algorithms. With the inclusion of such features, the overall system architecture becomes an intelligent DSS (iDSS), which exhibits high levels of flexibility, resilience, and trusted autonomy in several practical applications, also beyond EO. In future research, the suitability of iDSS to detect/analyse rare events in astronomy and astrophysics-based missions will be investigated. Furthermore, heterogeneous satellites and sensors will be considered, and effective scheduling and planning strategies will be examined for autonomous navigation and mission management tasks. As TASO capabilities evolve, humans will play a supervisory role in iDSS operations, shifting from human-in-the-loop to human-on-the-loop mission concepts. This will place emphasis on iDSS flexibility and resilience (i.e., the ability of such systems to properly react/adapt to both changing requirements and hardware/software malfunctions), enabling a timely and effective reconfiguration of the iDSS to successfully accomplish space missions with no or little performance degradations.

## Figures and Tables

**Table 1:** DSS architecture types. Adapted from [24].

DSS Architecture Type		Mission Goals	Cooperation	Homogeneity	Operational/Functional Independence
**Constellation**		Mission goals shared (Iridium, GPS)	Cooperation is required to support the mission goals	In general, homogeneous components, some differences possible(GPS generations)	Autonomous
**Formation**	**Trains**	Mostly independent, but could be shared	Cooperation from optional to required	Heterogeneous components	Autonomous
	**Cluster**	Mission goals shared	Cooperation is required to support mission goals	Homogeneous components	From autonomous to completely co-dependent
	**Leader/follower**	Mission goals shared	Cooperation from optional to required	Heterogeneous components	From autonomous to completely co-dependent
**Swarms**		Mission goals shared	Cooperation required tosupport mission goals	From homogeneous to heterogeneous components	From autonomous to completely co-dependent
**Fractionated**		Shared mission goals	From optional (service areas) to required (distributed critical spacecraft functions)	Heterogeneous components	From autonomous to completely co-dependent
**Federated**		Independent mission goals	Ad hoc, optional	Heterogeneous components	Autonomous
**Modular**		Mission goals shared	Cooperation is required to support mission goals	From homogeneous to heterogeneous components	From autonomous to completely co-dependent
**Hybrid**		Mostly independent, but could be shared	Ad hoc, optional	Heterogeneous components	From autonomous to completely co-dependent
**Constellation of formations**		Mostly shared but could be independent	Cooperation is required to support mission goals	From homogeneous to heterogeneous components	From autonomous to completely co-dependent

**Table 2:** List of optical sensors. Adapted from [61,62].

Satellite/Sensors	Number of Bands	Spatial Resolution (m)	Temporal Resolution (Day)	Swath (km)	Scale of Application	Data Availability
**NOAA/AVHRR**	5	1100	0.5	2800	R–G	1978
**MODIS**	36	250–1000	0.5	2330	R–G	1999
**Suomi NPP-VIIRS**	22	375–750	0.5	3040	R–G	2012
**MERIS**	15	300	3	1150	R–G	2002–2012
**Sentinel-3 OLCI**	21	300	2	1270	R–G	2016
**Landsat**	4–9	15–80	16	185	L–G	1972
**SPOT**	4–5	2.5–20	26	120	L–R	1986
**Aster**	14	15–90	16	60	L–G	1999
**Sentinel-2 MSI**	13	10–60	5	290	L–R	2015
**IKONOS**	5	1–4	1.5–3	11.3	L–R	1999
**QuickBird**	5	0.61–2.24	2.7	16.5	L	2001
**WorldView**	4–17	0.31–2.40	1–4	17.6	L	2007
**RapidEye**	5	5	1–5.5	77	L–R	2008
**ZY-3**	4	2.1–5.8	5	50	L–R	2012
**GF-1/GF-2**	5	1–16	4–5	800	L–R	2013
**PRISMA**	238	5–30	29 *	30	L–G	2019
**Hyperscout-2**	48	75–85	4-7	~300	L–G	2020

* Relook capability of 7 days with roll manoeuvre; L, landscape; R, regional; G, global; L–R, landscape to regional; L–G, landscape to global; R–G, regional to global.

**Table 3:** Hardware accelerators performance [27,57].

HW Accelerator	Inference Time (ms)	Power Consumption (W)
**Movidius**	5.8	1.4
**Jetson TX2**	3.0	4.8 (2.1 GPU only)
**Jetson Nano**	3.4	2.6 (2.0 GPU only)

**Table 4:** System-wide coverage parameters with respect to a scenario duration of 6 h, i.e., 21,600 s.

Location	Latitude (deg)	Longitude (deg)	n	SWAD (sec)	SWAP (%)	N	$SWAD_{T}$	$SWAP_{T}$
**Africa**	11.2027	17.8739	10	300	1.3889	545	16,350	75.6944
**Australia**	−31.25	146.92	5	150	0.6944	691	20,730	95.9722
**Europe**	40.1209	9.0129	10	300	1.3889	712	21,360	98.8889
**North America**	44	−120.3	14	420	1.9444	699	20,970	97.0833

## Data Availability

The code and data are available here: https://github.com/DarioSpiller/Tutorial_PRISMA_IGARSS_wildfires, accessed on 26 January 2023.
